# CD300a Receptor Blocking Enhances Early Clearance of *Leishmania donovani* From Its Mammalian Host Through Modulation of Effector Functions of Phagocytic and Antigen Experienced T Cells

**DOI:** 10.3389/fimmu.2021.793611

**Published:** 2022-01-18

**Authors:** Rajan Singh, Anshul Anand, Arun K. Rawat, Shashi Saini, Baishakhi Mahapatra, Naveen K. Singh, Alok K. Mishra, Samer Singh, Nisha Singh, Dhiraj Kishore, Vinod Kumar, Pradeep Das, Rakesh K. Singh

**Affiliations:** ^1^ Department of Biochemistry, Institute of Science, Banaras Hindu University, Varanasi, India; ^2^ Centre of Experimental Medicine and Surgery, Institute of Medical Science, Banaras Hindu University, Varanasi, India; ^3^ Department of Medicine, Institute of Medical Science, Banaras Hindu University, Varanasi, India; ^4^ Department of Molecular Biology, Rajendra Memorial Research Institute, Patna, India

**Keywords:** CD300a, macrophages, dendritic cells, T cells, *Leishmania*

## Abstract

The parasites of the genus *Leishmania* survive and proliferate in the host phagocytic cells by taking control over their microbicidal functions. The parasite also promotes differentiation of antigen-specific anti-inflammatory cytokines producing effector T cells, which eventually results in disease pathogenesis. The mechanisms that parasites employ to dominate host adaptive immunity are largely unknown. For the first time, we report that *L. donovani*, which causes visceral leishmaniasis in the Indian subcontinent, upregulates the expression of an immune inhibitory receptor i.e., CD300a on antigen presenting and phagocytic cells to dampen their effector functions. The blocking of CD300a signals in leishmania antigens activated macrophages and dendritic cells enhanced the production of nitric oxide, pro-inflammatory cytokines along with MHCI/II genes expression, and reduced parasitic uptake. Further, the abrogation of CD300a signals in *Leishmania* infected mice benefited antigen-experienced, i.e., CD4^+^CD44^+^ and CD8^+^CD44^+^ T cells to acquire more pro-inflammatory cytokines producing phenotypes and helped in the early clearance of parasites from their visceral organs. The CD300a receptor blocking also enhanced the conversion of CD4^+^ T effectors cells to their memory phenotypes i.e., CCR7^high^ CD62L^high^ up to 1.6 and 1.9 fold after 14 and 21 days post-infection, respectively. These findings implicate that CD300a is an important determinant of host phagocytic cells functions and T cells differentiation against *Leishmania* antigens.

## Introduction

*Leishmania donovani* belongs to the family of intracellular protozoan parasites of the genus *Leishmania* that causes visceral leishmaniasis (VL), which is fatal if left untreated (1). Since the identification of the causative agent, the pentavalent antimonial compounds had remained a mainstay treatment for all forms of leishmaniasis but the emergence of drug-resistant parasites has restricted their use in many endemic regions (2). Amphotericin B and miltefosine, the antifungal and antitumor compounds, respectively are recommended second-line drugs but their toxicities and the possibility of resistance development remain a serious looming threat worldwide (3, 4). Additionally, the efficacy of these two drugs is also being questioned for their inability to produce a sterile cure, which is evident by an unusual presentation of VL in the form of post kala azar dermal leishmaniasis (PKDL) even years after successful treatment (5). Recent studies suggest that individuals with PKDL are potential parasite reservoirs and may be a reason for disease surges in endemic and non-endemic regions (6, 7).

The existing knowledge on *Leishmania* immunobiology seems to be inadequate to design and develop an effective immunoprophylactic control measure. In the recent past, various vaccine candidates have been evaluated but all of them failed to produce the required threshold of protective immunity (8). Although, the protective immunity in leishmaniasis has remained a controversial issue, yet the existence of a large number of seropositive asymptomatic individuals in disease-endemic areas and their subsequent conversion to seronegative state, suggests that in all probability protective immunity against leishmaniasis exists (9). Thus, for the identification of potential protective immune correlates more studies are needed to explore the host-parasite relationship.

*Leishmania* is a naturally versatile parasite, equipped with highly efficient antioxidant defense machinery that helps in their survival and proliferation inside host phagocytic cells by dampening their microbicidal activities (10). In addition, the parasitic antigens also downregulate antigens presenting cells (APCs) effector functions like the expression of major histocompatibility complex (MHC) and co-stimulatory receptors, which diverts naïve T cells differentiation into anti-inflammatory cytokines secreting phenotypes that further exacerbate disease pathology (11–14). Traditionally, studies on parasitic infections largely explore immune activating i.e., immune response (IR) pathways in response to parasitic antigens but the acquired knowledge, so far, seems inadequate to design and develop newer prophylactic and therapeutic strategies. Conversely, our understandings on mechanisms of immune inhibition, which are mediated by the various ubiquitously expressed receptors on the surface of innate and adaptive immune cells like CD200, CD200R, TIM3, CD300a, is very limited (15, 16). The purpose of inhibitory mechanisms is to maintain a fine balance between immune activation and inhibition to protect the host from the harmful effects of exacerbated immune response along with restricting the possibilities of tolerance development in T cells against specific antigens (17). Recent observations on these receptors have revealed their extended regulatory roles in the acquisition of specific functional characteristics by phagocytic and T cells in tumor progression as well as in viral and bacterial pathogeneses (18–20). However, they are relatively less explored in parasitic infections.

In this work, we have explored the role of CD300a in the regulation of effector functions of macrophages, dendritic cells, and antigen experienced T cells in *L. donovani* infection. CD300 is a multigene family that has seven members named alphabetically (a-g) according to their location on chromosome 17q25 in humans whereas in mouse they are coded by nine genes located on chromosome 11 (21). These receptors have an extracellular (Ig) V-like domain with either immunoreceptor tyrosine-based activation (ITAMs) or inhibition (ITIMs) motifs as an intracellular tail for delivering activating (e.g., CD300b & CD300e) or inhibitory (e.g., CD300a & CD300f) signals, respectively (22, 23). The CD300a is ubiquitously expressed on both, myeloid and lymphoid cells, and after binding with ligands it facilitates the interaction with adaptor proteins phosphatidylinositol-3-kinase or ITIMS that relay inhibitory signals to abrogate the cellular activation (21). CD300a specifically recognizes phosphatidylserine (PS) and phosphatidylethanolamine (PE) as ligands, which are exposed on the outer surface of dead and activated cells (24). After phosphorylation of ITIMs, it recruits various phosphatases like src homology region 2 domain-containing phosphatase SHP 1 & 2, or Src homology region 2 containing inositol phosphatase (SHIP) depending on the cell types and their specific activating signals to deliver the inhibitory signals (25).

It has been found that CD300a regulates diverse signaling pathways of both innate and adaptive immune cells that control processes like cellular differentiation and viability (26, 27), cytokines/chemokines secretion (28), phagocytosis (29), inflammation (30, 31) and chemotaxis (32). Studies have also indicated that the CD300a receptor also helps in CD4^+^ T cells differentiation into Th1 phenotypes to produce pro-inflammatory cytokines (33, 34). However, its role in the modulation of immune cells effector functions is not studied in pathogenesis caused by protozoan parasites. Since *Leishmania* expresses plenty of CD300a ligands PS/PE in the outer leaflet of the cytoplasmic membrane, we launched this study to identify its regulatory role in the visceral form of leishmaniasis. The data surmised that the parasite increases CD300a receptor expression on phagocytic and dendritic cells for its growth and survival. Further, the abrogation of CD300a signals yielded more proliferative and functional antigen experienced T cells, and also promoted early resolution of clinical symptoms in the infected animals.

## Materials and Methods

### Animals and Parasites

Five to six-week-old female BALB/c mice were used in this study. Animals were housed in a pathogen-free animal house, fed with a normal routine diet and water *ad libitum*. All the procedures used in the study were reviewed and approved by the Ethics Committee for Animal Care and Use, Institute of Science, Banaras Hindu University, Varanasi (No.F.Sc./88/IAEC/2016-17/174). A cloned line of *L. donovani* parasites (MHOM/IN/1983/AG83) was used for the experiments and its virulence was maintained either in mice or hamsters. The promastigote forms of the parasite were cultured at 26°C in M199 media (pH 7.2-7.5) supplemented with antibiotics (penicillin, streptomycin, gentamycin), 0.1% hemin, 10 mM adenine, and 10% FBS. Mice were infected with stationary phase parasites (3x10^6^/30µl) intravenously *via* tail vein and loads of parasites in spleens were determined by serial dilution method as described elsewhere (35).

### Isolation of Lymphocytes From the Spleen and Peripheral Lymph Nodes

For T cells isolation, mice were sacrificed at the designated days of experiments to remove spleen and lymph nodes. The splenocytes were obtained by macerating spleens with a plunger and passing the tissue homogenates through a 70μm cell strainer. Cells were washed 2-3 times by centrifugation at 500g at 4°C for 5 min with incomplete RPMI-1640 to obtain a single-cell suspension. Pan-T cells were isolated using Pan-T cells isolation kit (Miltenyi Biotec, USA) using magnetic columns as per the manufacturer’s instructions. As and when required, the CD4^+^ and CD8^+^ T cells were isolated by positive selection using appropriate isolation kits (Miltenyi Biotec, USA). For isolation of lymph nodes (LN) lymphocytes, the brachial, axillary, and inguinal nodes were harvested and pooled together and the cells were isolated by mechanical maceration as described above.

### Macrophages and BMDCs Culture

RAW264.7 macrophages were obtained from NCCS Pune, India, and maintained in DMEM medium supplemented with FBS (10%) and antibiotics (penicillin/streptomycin-1%) at 37°C in a CO_2_ incubator in the atmosphere of 5% CO_2_ and 95% humidity. The dendritic cells were derived from mice femur and tibia bone marrow hematopoietic stem cells (HSCs) using recombinant IL-4 and granulocyte monocyte colony stimulating factor (GM-CSF). Briefly, the HSCs were cultured in complete RPMI-1640 medium supplemented with FBS (10%), antibiotics, IL-4, GM-CSF for 7-8 days in order to obtain differentiated bone marrow-derived dendritic cells (BMDCs). The purity of cells was determined by flow cytometry, and 80-90% pure CD11c^+^ DCs were used for all the experiments.

### Parasite Infection in Macrophages/BMDCs and Measurement of Parasitic Load

The regulatory roles of CD300a on phagocytes effector functions were first validated in *in-vitro* experiments. The cells, macrophages, and BMDCs, were infected with parasites in a ratio of 1:10 (cells to parasites) for 6h in a CO_2_ incubator. After incubation, the cells were thoroughly washed to remove non-internalized/phagocytosed parasites with warm incomplete media or PBS (0.02M, pH 7.2) and were further kept in the incubator for the desired time as per experimental needs. Cells were stained with Giemsa stain and intracellular amastigotes were counted under a microscope (Nikon Eclipse Ti-S, Japan). The parasite count was represented as amastigote number per 100 macrophages/BMDCs.

### Quantification of CD300a Expression in Macrophages/BMDCs and Measurements of Their Effector Properties

The mRNA and protein expression levels of CD300a were quantified by qPCR and immunoblotting, respectively in infected and non-infected cells. The effector properties of macrophages and BMDCs were quantified in terms of their capacity to produce nitric oxide, pro and anti-inflammatory cytokines, and MHC genes expression. We estimated nitrite (NO_2_^-^) species in the culture supernatant, which was used as an indicator of nitric oxide (NO) production. The nitrite species were quantified using Griess reagent as described elsewhere (36). In brief, 100µl of supernatant was mixed with freshly prepared Griess reagent (1% sulfanilamide, and 0.1% naphthyl ethylene diamine in 5% phosphoric acid). The mixture was incubated for 15–20 min at room temperature, and the absorbance was recorded at 540 nm on an ELISA plate reader (Bio-Rad, USA). The expression levels of MHC I/II were quantified either by qPCR or immunoblotting. Levels of secretory cytokines were quantified by cytokine ELISA kit (Ray Biotech, USA) and qPCR. The details of qPCR and immunoblotting are described in the later sections. All *in vitro* measurements were made at 12h, 24h, 36h, or 48h post infections unless mentioned otherwise in the figure legends.

CD300a receptors on macrophages and BMDCs were blocked prior to *Leishmania* infection using monoclonal anti-mouse CD300a antibodies (Cat#MA5-23927, Invitrogen, USA, IgG2a, 172224, RRID: AB_2607219) to delineate its regulatory role. Briefly, cells were activated (as and when required) with soluble leishmanial antigens (SLA; 10µg/ml) and kept overnight in a CO_2_ incubator. The next day, anti-CD300a antibodies were added (10µg/ml) to the activated cells and further incubated for 4h before infection. After incubation cells were washed and infected with parasites as described above. The measurement of effector functions parameters was made as per experimental needs as described above. The cells treated with isotype antibodies (Cat#14-4321-82, Invitrogen, USA, IgG2a, eBR2a, RRID: AB_470105) were used as control. To check the effect of anti-CD300a antibodies on parasitic growth/proliferation, the parasites were cultured in the presence of antibodies (1µg/10^8^ parasites), and their proliferation was measured by MTT assay at 4h, 24h, 48h, and 72h. The parasites treated with a high-affinity PE binding antibiotic duramycin (50µg/100µl/10^8^ parasites) were used to validate if blocking of PE on the *Leishmania* surface reduces their uptake. The parasites were incubated with duramycin for 10 min and washed 2-3 times. Subsequently, the treated parasites were cultured, and parasitic growth was determined after 24h.

### BMDCs and T Cells Co-Culture Studies: Measurement of CD4^+^ and CD8^+^ T Cells Proliferative and Cytokines Producing Abilities

The role of CD300a on the effector properties of antigen-experienced CD4^+^/CD8^+^ CD44^+^ T cells was first validated in *in-vitro* experiments. The infected BMDCs were cocultured with antigen-experienced pan T cells that were obtained from *Leishmania* infected animals post 14 days of infection. BMDCs were first activated with SLA overnight, washed and blocked with anti-CD300a antibodies, and then infected with parasites as described above. After removal of non-phagocytosed parasites, the pan T cells were added to the cultured BMDCs at a ratio of 10:1 (cells to BMDCs or macrophages).

For proliferation studies, cells were stained with 2.5µM CFSE (Invitrogen, ThermoFisher, USA) for 10 min in RPMI 1640 medium without FBS. The extracellular CFSE quenching was done by adding ice-cold complete medium (RPMI 1640 plus 10% FBS) to the stained cells and after 5min cells were washed before adding to cultured BMDCs. Cells were cultured for 5 days at 37°C in a CO_2_ incubator under 5% CO_2_ atmosphere and their relative proliferation was assessed by flow cytometry. The cytokine production abilities of antigen-experienced CD4^+^ and CD8^+^ T cells were quantified by flow cytometry as described in the surface and intracellular surface staining section. Alternatively, CD4^+^ and CD8^+^ T cells were co-cultured with anti-CD300a treated parasite-infected macrophages and their differential proliferation was determined by MTT assay (37).

### CD300a Receptor Expression Quantification and Blocking in Mice

The relative expression of CD300a was measured at days 7, 14, and 21 post-infections in CD300a blocked and unblocked animals. The parasite infectivity was confirmed by splenic parasitic load and spleen size. The quantitation of CD300a mRNA transcripts and proteins level in the spleen was done by qPCR and immunohistochemistry, respectively. Additionally, CD300 expression on the splenic CD11C^+^ dendritic cells was also measured by flow cytometry. For evaluating the effect of CD300a blocking on different aspects of VL pathogenesis i.e., parasite growth, effector functions of antigen-experienced CD4^+^/CD8^+^ T cells, and their conversion into memory phenotypes during early immune responses, the mice were treated with anti-CD300a antibodies (10µg/kg of body weight) intravenously at days 0, 2, 4, 6 post-infections. The control group received isotype antibodies as and when required. At the desired time points, mice were sacrificed to measure splenic parasite load and functions of antigen-experienced CD4^+^/CD8^+^CD44^+^ T effectors cells. The CD4^+^ T cells expressing memory markers i.e., CCR7^+high^ and CD62L^+high^ were quantified in lymph nodes of anti-CD300a antibody-treated and untreated mice.

### Measurement of mRNA Transcripts and Protein Levels

The mRNA expression levels of CD300a, MHC I/II, and cytokines (pro and anti-inflammatory) genes were quantified by qPCR. The primers used in this study are listed in **Supplementary Table 1**. Total RNA was extracted from cells (macrophages/BMDCs/splenocytes) and spleen tissues using TRI^®^ reagent (Sigma-Aldrich, USA). The cDNA was synthesized from 1µg of total RNA using a cDNA synthesis kit (Applied Biosystems, USA) as per the manufacturer’s protocol. The cDNA was amplified on Applied Biosystems 7500 Fast Detection system with SYBR green qPCR master mix as per manufacturer’s instructions (Applied Biosystems, USA). All reactions were performed in triplicate and negative controls (no template cDNA) were included in each experiment. GAPDH was taken as internal control and all the data sets were normalized to the level of GAPDH (38). Fold change in gene expression was calculated by the Δ^2^CT method and results were reported as arbitrary units or fold changes.

For the relative quantification of CD300a and MHC I/II proteins, the cells or spleen tissues were lysed in 300μl lysis buffer (50mM Tris-HCl, pH 8.0, 150mM NaCl, 1% Nonidet P-40, 0.50% sodium deoxycholate, 0.10% SDS) containing protease inhibitor cocktail (Sigma-Aldrich, USA) and centrifuged at 10,000g for 15 min at 4°C to remove cell debris and other insoluble materials. The lysate proteins (50μg/lane) were resolved on SDS-PAGE (10%) and transferred to nitrocellulose membrane using Trans-Blot Turbo (BioRad Laboratories, USA). Membranes were incubated in blocking buffer (2.5% BSA, 20% Tween-20 in PBS) for 4h, which was followed by PBS wash and further incubation for 4h with anti-CD300a antibodies at room temperature (RT). Finally, membranes were incubated with HRP-coupled anti-rabbit IgG or anti-mouse IgG secondary antibodies, and blots were developed using ECL reagent. Digital quantification of chemiluminescence was performed using Image J software having β-actin as control (NIH, USA) (39).

### Immunohistochemical Measurement of CD300a Expression

The CD300a expression in the spleen of infected animals was also quantified by immunohistochemistry. Animals were first anesthetized with diethyl ether and perfused intracardially with chilled 0.9% saline and 4% paraformaldehyde, which was prepared in 0.01M PBS (pH7.4). Spleens were kept in 10% paraformaldehyde overnight and further transferred to 10% sucrose solution before immunohistochemical staining for CD300a. Tissue sections of 20µm thickness were cut coronally using a cryomicrotome. The sections were washed with 0.01M PBS (pH7.4) at 10 min intervals and then blocked with 10% normal goat serum (NGS) containing 0.3% Triton-X 100 and 1% BSA for about 1h. After washing, the sections were incubated with R-PE tagged monoclonal anti-mice CD300a antibody (Miltenyi Biotec, LMIR1-PE, RRID: AB_2657121) at 1:1000 dilutions for 16h at 4°C. The sections were washed with PBS containing 1% BSA four times to remove unbound antibodies and then DAPI was added at a final concentration of 1µg/ml. After brief incubations, the sections were washed thrice with PBS and then mounted on slides using polyvinyl alcohol mounting medium containing DABCO anti-fading agent (Fluka Analytical, USA). The images were taken by confocal microscope (Carl Zeiss, Germany). Relative immunofluorescence was estimated by Image J software and reported as the mean integrated fluorescent values of CD300a expression in the spleen.

### Liver Granuloma Staining

For liver granuloma staining, organs were isolated in 10% paraformaldehyde and fixed in Bouin’s fluid for 24h. Before processing for tissue sections, the organs were washed with different concentrations of ethanol and were kept for 5min in xylene just before preparation of wax specimen blocks. The 5μm thickness microsections were stained with hematoxylin-eosin (H&E) as per standard protocol. Photomicrographs were taken at 20x magnification under a light microscope to analyze granuloma formation.

### Flow Cytometry: Surface and Intracellular Staining

The surface and intracellular staining for phenotypic characterization and quantification of cytokines producing abilities of CD4^+^/CD8^+^CD44^+^ T effectors (T_eff_) cells were measured on days 7, 14, 21 post-infection. Before surface staining, Fc receptors on cells surface were blocked with rat anti-mouse CD16/32 (1µg/10^6^ cells) antibodies for 20 min. Cells were surface stained with anti-CD3-eFluor506 (Cat# 69-0032-82, RRID: AB_2637122), anti-CD4-Superbright702 (Cat#67-0042-82, RRID: AB_2662399), anti-CD8-Superbright702 (Cat# 67-0081-82, RRID: AB_2662351), anti-CD44-Superbright436 (Cat# 62-0441-82, RRID: AB_2573520), anti-CD300a-PE (Cat# 130-109-036, RRID: AB_2657121), anti-CCR7-APCeFluor780 (Cat# 47-1971-82, RRID: AB_2573974) and anti-CD62L-PE-Cyanine7 (Cat# A14721, RRID: AB_2534237) antibodies as per manufacturer’s instructions for 30 min at 4°C or ice. For intracellular cytokines staining, cells were washed and fixed for 20 min at RT using the cytofix/cytoperm kit (ThermoFisher, USA). Intracellular staining was done with anti-IFNγ-APC-eFluor780 (Cat# 47-7311-82, RRID: AB_2688061), anti-IL-12-eFluor660 (Cat# 50-7123-82, RRID: AB_11218493), and anti-IL-10-Alexa Fluor700 (Cat# 56-7101-82, RRID: AB_891568) antibodies for 30 min at 4°C or ice. Anti-CD300a-PE was purchased from Miltenyi Biotec, USA. All antibodies were purchased from the Invitrogen, ThermoFisher, USA. Cells were acquired on Attune NxT Flow cytometer equipped with required laser lines (BRV) using Attune NxT software. Specific cell populations were identified according to specific fluorescent-labeled antibodies and flow cytometry analysis was performed with the acquisition of minimum 3×10^6^ events per experiment. Data were analyzed with FCS Express™ version 7.1 (*De Novo* Software, Los Angeles, CA). Wherever needed, the cells were activated with SLA to induce intracellular cytokines production before analysis. The live-dead staining was done using LIVE/DEAD Fixable Aqua (Invitrogen, ThermoFisher, USA) to mark dead cells.

### Statistical Analysis

All *in-vitro* experiments were done in triplicate and repeated twice or thrice. In each animal group, six to eight mice were used. Real-time PCR data was calculated by the Δ^2^CT method and presented as fold change of genes expression levels. All the statistical analyses were performed on GraphPad Prism 7.0 software. The parametric tests (Students t-test or ANOVA) were performed to calculate the significance levels between means of groups and a p-value <0.05 was considered significant. All data are presented in mean ± SEM i.e., standard error of the mean.

## Results

### *L. donovani* Upregulates CD300a Expression in Cultured RAW 264.7 Macrophages, BMDCs, and in the Splenic CD11c^+^ Dendritic Cells and Tissues

**Figure 1:**
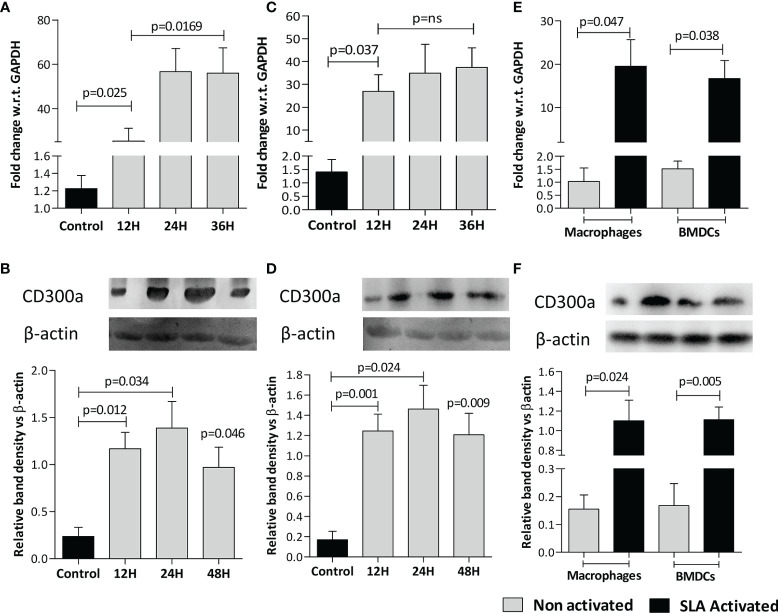
*L. donovani* infection and leishmanial antigen exposure induce CD300a expression in macrophages (Mφ) and BMDCs. The CD300a expression both, at mRNA and protein levels invariably increased in Mφ-like cell line RAW 264.7 **(A, B)** and BMDCs **(C, D)** on infection with parasites (at 1:10 ratio for 6h) at all time points tested post infection as compared to uninfected control. The soluble leishmanial antigens (SLA) also significantly induced CD300a expression in uninfected macrophages and dendritic cells at both mRNA (Mφ p=0.047; BMDCs p=0.038; **E**), and protein (Mφ p=0.024; BMDCs p=0.005; **F**) levels as compared to nonactivated cells.

The expression of CD300a both, at mRNA (**Figures 1A, C**) and protein (**Figures 1B, D**) levels were found significantly increased in macrophages (Mφ) and BMDCs infected with *L. donovani* at all the time points, i.e., 12h, 24h, 36h being maximum at 24h post-infection. The activation of cells with soluble leishmanial antigens (SLA) also increased CD300a mRNA (Mφ,23.8 fold, p= 0.047; BMDCs, 13.7 fold, p=0.038) and protein (Mφ, 7 fold, p=0.024; BMDCs, 6.6 fold, p=0.005) expression levels that was measured 24h post-infection (**Figures 1E, F**). The parasites infectivity, represented as the number of amastigotes in infected macrophages and BMDCs at different time points, are presented in **Supplementary Figures 1A, B**, respectively. We did not observe any impact of anti-CD300a monoclonal antibodies on the growth of the parasites (**Supplementary Figure 1C**). The blocking of parasite surface PE with duramycin reduced parasite uptake in both, macrophages (1.8 fold; p=0.018) and dendritic cells (1.6 fold; p=0.023), suggesting a role of PE in parasitic uptake (**Supplementary Figure 1D**). In addition, we did not observe any difference in growth rates of duramycin treated and untreated parasites, measured at 24h, which is depicted in the inset of **Supplementary Figure 1D**.

**Figure 2:**
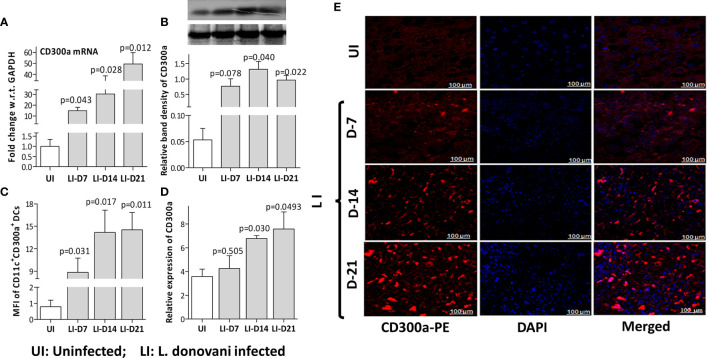
The CD300a mRNA and protein expression levels in the spleen of L. donovani infected mice. Mice (n=6 per group), infected with 3×10^6^ stationary phase promastigotes *via* tail vein, were evaluated post 7 (D-7), 14 (D-14), and 21 (D-21) days of infection. The expression levels of CD300a mRNA **(A)** and protein **(B)** were significantly elevated in infected mice with the passage of time as compared to day 7. **(C)**. The number of CD300a expressing CD11c+ dendritic cells in the spleen, measured as Mean Fluorescent Intensity (MFI) by flow cytometry, were found significantly increased at all time points post-infection. An unvarying expression of CD300a was observed in the spleen tissues **(E)** that increased with the passage of time in the infected animals **(D)**. The uninfected (UI) animals were used as a control for comparison to determine statistical significance (p-value).

To validate CD300a’s role in the survival of parasites during the early immune responses mounted by the host, we quantified the expression levels of CD300a in mice spleens at days 7 (D7), 14 (D14), and 21(D21) post-infection. A gradual increase at both, mRNA (D7, p=0.043; D14, p=0.028; D21, p=0.012) and protein (D7, p=0.078; D14, p=0.040; D21, p=0.022) levels of CD300a was observed in the spleen of infected mice (**Figures 2A, B**). The frequency of CD300a^+^ CD11c^+^ dendritic cells was also found to be increased by 11-18 fold (D7, 11.2 fold, p=0.031; D14,18.5 fold, p=0.017; D21, 18.4 fold, p=0.011) after infections (**Figure 2C**). The immunohistochemical observations also corroborated the above findings (**Figures 2D, E**). These initial findings prompted us to further evaluate the role of CD300a in shaping early immune response against *L. donovani*.

### Blocking of CD300a Receptor Enhanced Effector Functions of Macrophages and BMDCs

**Figure 3:**
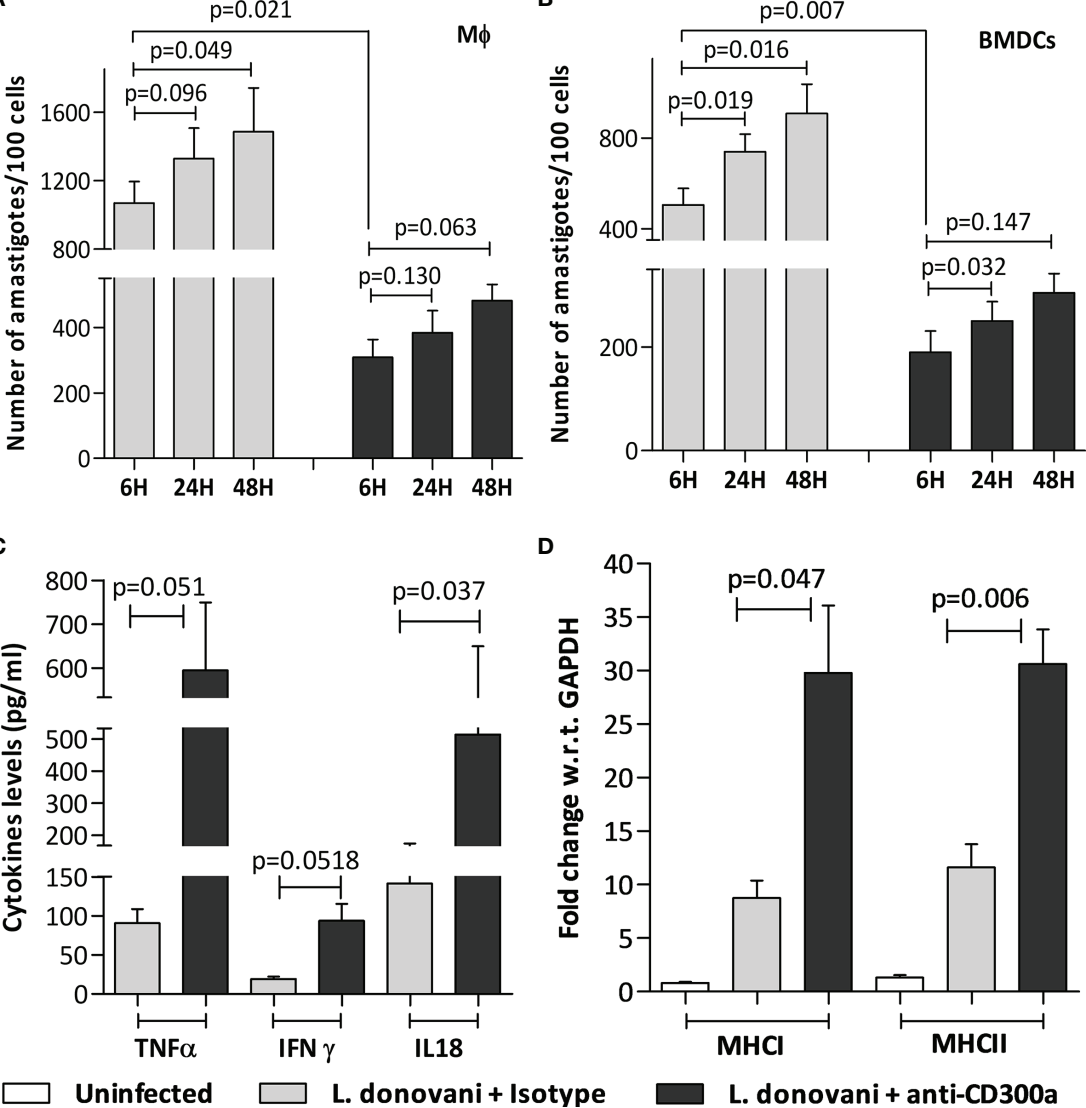
Parasite load, cytokine levels, and MHC genes expression in anti-CD300a antibodies treated and untreated Mφ and BMDCs. **(A, B):** The prior incubation with anti-CD300a antibodies resulted in the reduced uptake of parasites in the SLA activated Mφ-like cell line RAW 264.7 (p=0.021; **A**) and BMDCs (p=0.007, **B**). The amastigote proliferation after 24h and 48h was found similar in cells in treated and untreated groups. The extracellular levels, measured after 72h by cytokine ELISA, of pro-inflammatory cytokines. TNF-α (p=0.051), IFN-γ (p=0.051), and IL-18 (p=0.037) **(C)** along with MHC I and II genes expression levels **(D)** were elevated in SLA activated and parasites infected BMDCs after anti-CD300a antibodies treatment.

We hypothesized that induced expression of CD300a on antigen-presenting cells may have a significant impact on effector properties of activated T cells during infection. To validate this hypothesis, we first evaluated the role of CD300a blocking on the effector properties of antigen-presenting cells. Cells were treated with anti-CD300a antibodies after SLA activation and then infected with *Leishmania* parasites. The anti-CD300a antibodies treatment reduced parasitic uptake in both, macrophages (3.4 fold; p=0.021) and BMDCs (2.6 fold; p=0.007) as compared to untreated cells albeit their proliferation rate seemed similar in both cells (**Figures 3A, B**). Next, we measured the pro-inflammatory cytokines production abilities of dendritic cells after the blocking. The levels of TNF-α (6.5 fold, p=0.051), IL-18 (4.8 fold, p=0.037) and IFN-γ (3.6 fold, p=0.051) were found significantly elevated in anti-CD300a treated cells (**Figure 3C**). The CD300a receptor blocking also enhanced the expression of MHC I (1.5 fold; p=0.047) and MHC II (3.2 fold; p=0.006) genes in the infected BMDCs as compared to isotypic controls (**Figure 3D**). Further, the nitric oxide levels were also found significantly enhanced after the CD300a receptor blocking in both, macrophages (1.7 fold; p=0.025) and dendritic cells (2.6 fold; p=0.002) (**Supplementary Figure 2A**). These results suggested that anti-CD300a antibodies treatment enhances the effector properties of antigen presenting cells.

We also measured pro and anti-inflammatory cytokines levels in antibodies treated and untreated macrophages. An increase in the production of pro-inflammatory cytokines, i.e., IFN-γ (1.5 fold, p=0.098), TNF-α (1.7 fold, p=0.008), IL-12 (1.26 fold, p=0.16) and a decrease in anti-inflammatory cytokines, i.e., IL-4 (2.7 fold, p=0.001) and IL-10 (2 fold, p=0.020) levels were observed after anti-CD300a antibodies treatment (**Supplementary Figures 2B, C**), which further suggested an important regulatory role of CD300a receptor in *L. donovani* infection.

### *In Vitro* Blocking of CD300a Receptor on BMDCs Enhanced Antigen-Experienced CD4^+^ and CD8^+^T Cells Proliferation and Proinflammatory Cytokines Production

**Figure 4:**
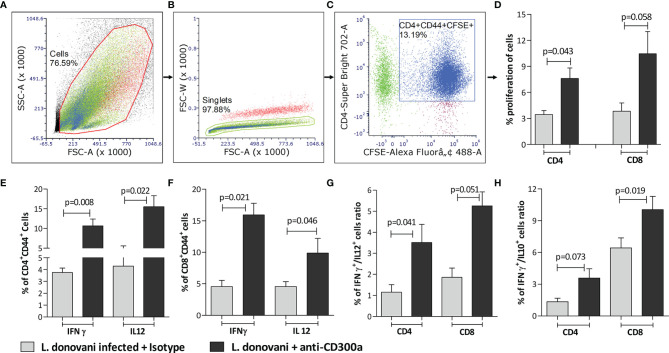
The proliferation rate and cytokines production of antigen experienced CD4^+^ and CD8^+^ T cells co-cultured with anti-CD300a antibodies treated and parasites infected BMDCs. To quantify T cells proliferation, the CFSE stained pan T cells isolated from splenocytes of animals after 14 days post-infection, were cultured with BMDCs (1:10; DCs to T cells ratio). A representation of gating strategies to quantify the percent of antigen experienced CFSE^+^ CD4^+^/CD8^+^ T cells is depicted in panels **(A-C)**. The CD4^+^ and CD8^+^ T cells cultured with anti-CD300a treated and parasite infected BMDCs were found more proliferative in comparison to the cells cultured with untreated BMDCs **(D)**. The CD300a receptor blocking significantly increased the production of IFN-γ and IL-12 in antigen experienced T cells **(E, F)**. The blocking of CD300as receptors also enhanced IFN-γ^+^/IL-12^+^ producing T cells **(G)**, and improved their IFN-*γ*/IL-10 ratio **(H)**. The cytokines positive T cells were measured on CD4^+^CD44^+^ T cells that were gated on CD3^+^ T cells and isotype antibodies were used as a control to determine statistical significance.

Next, we validated if an induced expression of CD300a on APCs affects the proliferation and functions of CD4^+^ and CD8^+^ T cells. We first did an *in vitro* experiment in which pan T cells (containing both CD4^+^ and CD8^+^ T cells), obtained from parasite-infected animals post 14 days of infection, were co-cultured with anti-CD300a antibodies treated and parasite infected BMDC. After antibodies treatment, the number of CD4^+^CD44^+^ and CD8^+^CD44^+^ T cells was increased up to 2.5 (p=0.043), and 3.2 fold (p=0.058), respectively (**Figures 4A–D**). A very similar enhancement in the number of CD4^+^ and CD8^+^ T cells cocultured with anti-CD300a antibodies treated and parasites infected macrophages was also observed (**Supplementary Figure 3**). Further, the CD300a blocking also enhanced the proportions of IFN-γ (CD4^+^, 2.8 fold, p=0.008; CD8^+^, 3.5 fold, p=0.021), IL-12 (CD4^+^, 3.6 fold, p=0.022; CD8^+^, 2.1 fold, p=0.046) positive cells (**Figures 4E, F**). The increased numbers of double-positive (IFN-γ/IL-12) CD4^+^ T cells (3 fold; p=0.041) and CD8^+^ T cells (2.83 fold; p=0.051) further suggested that the inhibition of CD300a signals on APCs enhances multiple cytokines producing abilities of antigen experienced T cells (**Figure 4G**). Further, IFN-γ/IL-10 ratio of CD4^+^ (p=0.073) and CD8^+^ (p=0.019) T cells were also found improved after CD300a receptor blocking (**Figure 4H**). These findings suggested that blocking of CD300a receptors on APCs helps antigen-experienced T cells to produce pro-inflammatory cytokines.

### CD300a Receptor Blocking Reduced Splenic Parasite Load, Abrogated Pathogenesis, and Helped Antigen-Activated CD4^+^/CD8^+^T Cells to Acquire Th1 Phenotypes in Infected Mice

**Figure 5:**
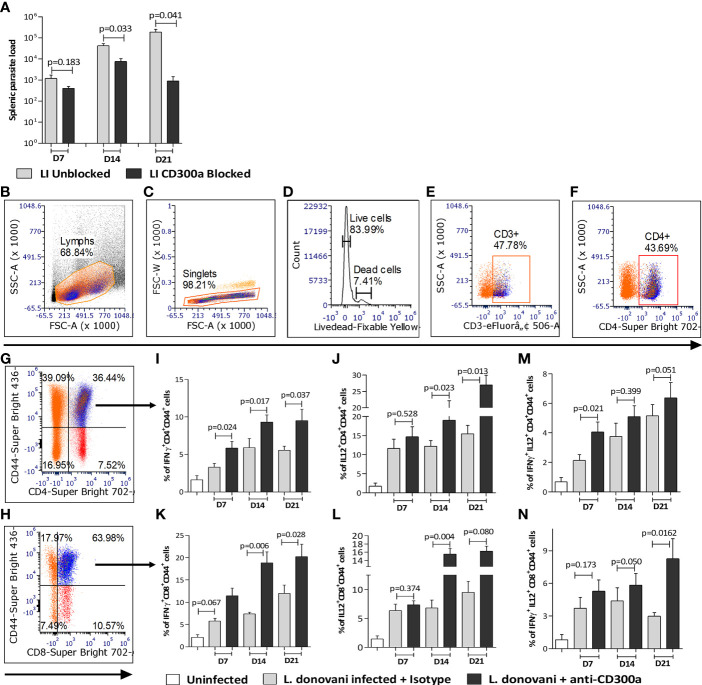
Spleen parasite load and pro-inflammatory cytokines producing antigen experienced CD4^+^ and CD8^+^ T cells in anti-CD300a antibodies treated mice. **(A)** The CD300a receptor blocking significantly reduced (at D14 and D21) splenic parasite load. **(B-H)** Flow cytometry gating strategies to select antigen experienced CD4^+^ and CD8^+^ T cells. The CD4^+^ and CD8^+^ T cells were gated on CD3^+^ T cells, which were selected on live cells as shown in panels **(B-F)**. The cytokines secreting CD4^+^ and CD8^+^ T cells were gated on CD4^+^CD44^+^ **(G)** and CD8^+^CD44^+^ T cells **(H)**. In CD300a blocked animals, the CD4^+^ T cells expressing IFN-γ^+^ **(I)**, IL-12^+^ **(J)**, and CD8^+^ T cells expressing IFN-γ^+^ **(K)**, IL-12^+^ **(L)** were significantly increased. The IFN-γ and IL-12 double-positive, CD4^+^, and CD8^+^ T cells were also increased in antibody treated animals **(M, N)**. The isotype antibodies treatment group was used as a control to determine statistical significance.

To validate the impact of CD300a signaling on the effector properties of T cells in *Leishmania* infected animals, we administered anti-CD300a antibodies to mice at the time of infection, i.e., at day 0 followed by three more doses at days 2, 4, and 6 post-infection and mice were sacrificed at days 7, 14 and 21 to record pathological and immunological observations. The isotype antibodies were used as a control to establish the explicit role of CD300a antibodies. The anti-CD300a antibodies treatment resulted in the reduced splenic parasite burden (D7, 2.9 fold, p=0.183; D14, 5.5 fold, p=0.033; D21, 208.3 fold, p=0.041) in infected mice as compared to those who received isotype antibodies (**Figure 5A**). The reduction in the splenic parasite load was not significant at D7 however, at D21 it reduced drastically (208 fold).

Further, the percent of CD4^+^CD44^+^ T cells expressing IFN-γ (D7, 1.7 fold, p=0.024; D14, 1.5 fold, p=0.017; D21, 1.7 fold, p=0.037; **Figure 5I**) and IL-12 (D7, 1.2 fold, p=0.528; D14, 1.5 fold, p=0.023; D21, 1.7 fold, p=0.013; **Figure 5J**), and CD8^+^CD44^+^ T cells expressing IFN-γ (D7, 1.9 fold, p=0.067; D14, 2.5 fold, p=0.006; D21, 1.7 fold, p=0.028; **Figure 5K**) and IL-12 (D7, 1.2 fold, p=0.374; D14, 2.2 fold, p=0.004; 1.7 fold, D21, p=0.080; **Figure 5L**) was significantly higher in the anti-CD300a treated mice than the untreated ones. Since multiple cytokines producing capabilities of T cells is a main determinant of efficient and protective T cells mediated immune response, we quantified IFN-γ/IL-12 expressing T cells in the CD300a blocked and unblocked mice. The percent of double-positive (IFN-γ^+^/IL-12^+^) CD4^+^ (D7, 1.9 fold, p=0.021; D14, 1.4 fold, p=0.399; D21, 1.3 fold, p=0.051; **Figure 5M**) and CD8^+^ (D7, 1.4 fold, p=0.173; D14, 1.3 fold, p=0.050; D21, 2.8 fold, p=0.0162; **Figure 5N**) cells were comparatively higher in CD300a antibodies treated mice, which further suggested that the antigen-experienced T cells acquire more multifunctional characteristics in absence of CD300a signals. The gating strategy to select antigen experienced T cells is presented in the **Figures 5B–H**.

Further, our findings also indicated that abrogation of CD300a signaling creates an inflammatory environment in the spleen of infected mice. It was corroborated by the increased levels of pro-inflammatory cytokines like IL-12, IFN-γ (**Supplementary Figures 4A, B**) and reduced levels of anti-inflammatory cytokines like IL-4 and IL-10 (**Supplementary Figures 4C, D**) at all three-time points (D7, D14, D21) of measurement. In addition, the expression levels of MHC I and MHC II genes were also found significantly elevated with increasing duration of infection in anti-CD300a treated mice (**Supplementary Figures 4E, F**). We observed a reduced number of granulomatous areas, specifically at the later stages of infection in the liver of anti-CD300a blocked mice, which further suggested its importance in controlling diseases outcome (**Supplementary Figure 5**). These observations provided strong evidence that intervention at the level of CD300a receptor could be a key to resolving *Leishmania* infectivity and pathogenesis.

### Blocking of CD300a Receptor Enhanced the Percent of CCR7^+^CD62L^+^CD4^+^ T Cells in Infected Mice

**Figure 6:**
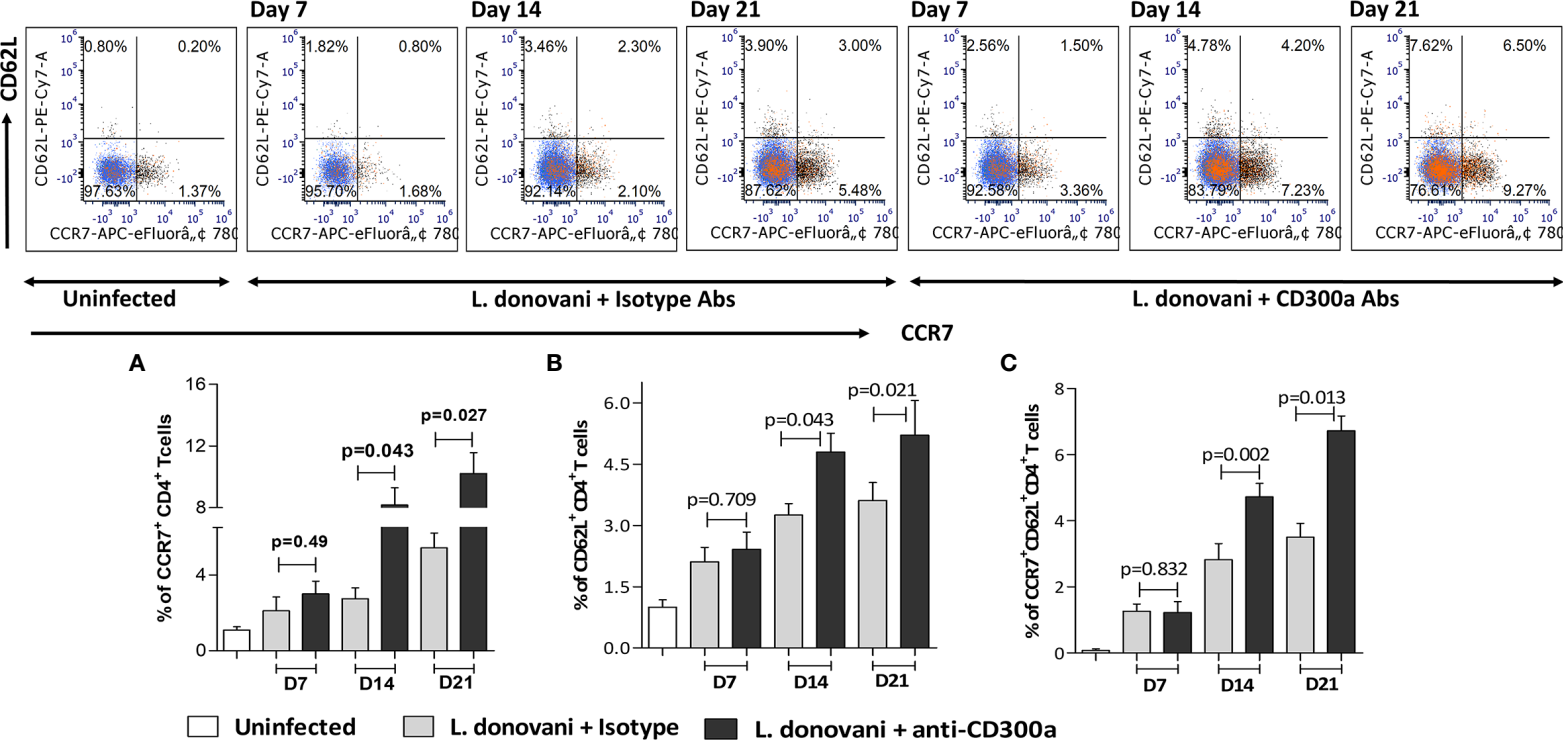
The memory phenotypes, i.e., CCR7^+^CD62L^+^CD4^+^ T cells in lymph nodes after CD300a blocking in infected animals. The number of CD4^+^ T cells expressing effector and central memory surface markers i.e., CCR7^+^ **(A)**, CD62L^+^ **(B)** along with double positives CCR7^+^CD62L^+^ central memory CD4^+^ T cells **(C)** were significantly higher at D14 and D21 in antibodies treated animals as compared to those who received isotypic antibodies.

An efficient immune response against an immunogen, which is characterized by highly proliferative and functional T cells, not only helps in early clearance of immunogen but also increases the possibility of effector T cells conversion into their memory phenotypes. Since we observed increased effector functions of antigen experienced CD4+ T cells post CD300a receptor blocking, therefore, we were interested to know if this also increases their conversion into their memory phenotypes. We quantified the central memory population of CD4^+^ T cells, expressing CCR7 and CD62L receptors on their surface in draining lymph nodes of infected mice. The CD4^+^ T cells expressing CCR7^high^ (D7, p=0.49; D14, p=0.043; D21, p=0.027; **Figure 6A**) and CD62L^high^ (D7, p=0.709; D14, p=0.043; D21, p=0.021; **Figure 6B**) were found increased in anti-CD300a antibodies treated mice that was significant at the later days i.e., at D14 and D21. The double-positives i.e., CCR7^high^CD62L^high^ CD4^+^ T cells (D7, p=0.8321; D14, p=0.002; D21, p=0.013) were also increased to about 1.6 fold at D14, and 1.9fold at D21 in blocked mice suggesting a possible role for CD300a signaling in the memory development against *L. donovani* antigens (**Figure 6C**).

## Discussion

Therapeutic strategies involving antibodies against immune inhibitory receptors like PD1, CTLA4, CD200 have been found very beneficial in preventing the progression of specific cancers through augmentation of T cell-mediated immunity (40, 41). The success of these strategies has paved a new pathway to develop alternate therapeutic, preventive or prophylactic, strategies for other diseases as well in which host immunity is compromised. CD300a is relatively a new addition to the family of inhibitory receptors and has been found to play important regulatory roles in viral pathologies (42), T cells differentiation and functions (33, 34) and in acute and chronic inflammations (43, 44). For the first time, we report CD300a as an important determinant of *Leishmania* pathogenesis by which parasite controls the effector functions of host phagocytic, antigen-presenting, and T cells. Our findings revealed that the parasite upregulates CD300a receptors expression on the surface of macrophages and dendritic cells to dampen their effector properties for the ease of its survival in the host cells. The abrogation of CD300a signaling enhances the nitric oxide, pro-inflammatory cytokines, and MHC I/II genes expression in phagocytic and antigen presenting cells. Further, we observed more proliferative, polyfunctional, and pro-inflammatory cytokines producing CD4^+^ and CD8^+^ T cells in the anti-CD300a antibodies treated mice in addition to early elimination of parasites from their visceral organs. These data suggest a very important role for the CD300a receptor in controlling *L. donovani* growth in the infected mice through modulation of the effector functions of antigen presenting and T cells.

The clinical outcomes of *Leishmania* infection depend on the early clearance of parasites by the phagocytic cells, which requires the support of T cells mediated immunity (45, 46). The phenotypic differentiation of T cells against parasitic antigens is an important factor that eventually determines resistance or susceptibility in all forms of leishmaniasis (47). During infection, the generation of Th2 phenotypes, which produce anti-inflammatory cytokines like IL-10, IL-4, TGF-β, is linked to susceptibility whereas induction of pro-inflammatory cytokines producing Th1 phenotypes confers resistance against infection (48, 49). In spite of significant research progress during the last couple of decades, the precise mechanisms or factors that regulate the phenotypic differentiation of naïve T cells are not identified in any form of leishmaniasis. This study reveals an important regulatory role of CD300a in shaping T cells function and thus, forms a basis to exploit its role in potentiating the antigenicity of parasitic antigens.

Although, the activation of naïve T cells during APC-T cells cross-talk is multifactorial, yet the nature of the antigen and its presentation to naive T cells are the main determinants of differentiation and function (50). The intracellular parasites and their antigens suppress MHC genes activation and other co-stimulatory molecules of APCs, which eventually lead to poor activation and altered functions of T and B cells that help in parasites survival (51, 52). The failure of *Leishmania* antigens as vaccine candidates generally stems from their inability to induce the required expression threshold of MHC genes necessary for appropriate activation of T or B cells, eventually leading to poor generation of memory cells and compromised protective immune responses. A study revealed that due to very limited capacity to induce the required threshold of MHC II and other co-stimulatory molecules like CD40 and CD86 by the dendritic cells, the kinetoplast membrane protein 11 (KMP-11), which was considered a potent vaccine candidate, failed to produce a durable protective immune response (53). Therefore, it’s a necessity to unravel the underpinnings of the APCs-T cells crosstalk to manipulate T cells differentiation, proliferation, and function against *Leishmania* antigens, in particular and parasitic antigens, in general. The inflammatory microenvironment during antigen presentation helps APCs to acquire their distinctive antigen-presenting abilities, characterized by the enhanced expression of MHCs and other costimulatory molecules on their surface, which determine the fate of naïve T cells against an antigen. A study on *Leishmania* documents that blocking of PS on the parasite surface helps dendritic cells to acquire inflammatory properties that eventually result in enhanced CD4^+^ T cells proliferation (54). We also observed enhanced MHC proteins and pro-inflammatory cytokines levels in the blocked BMDCs that were found linked with enhanced antigen experienced T cells function further suggesting that CD300a is a vital controller of T cells activation during APCs-T cells crosstalk.

The activated T cells that have the ability to acquire homing receptors like CCR7 and CD62L on their surface make up a very distinct population i.e., central memory. They home to lymph nodes as antigen specific T cells reservoirs and behave exactly similar to the effector T cells after secondary antigen exposure (55). In general, T cells response during early infective stages comprises both effector and memory phenotypes (56), and the APC-T cells crosstalk plays a decisive role in determining the numbers and the fate of central memory cells against an antigen (57, 58). Though the factors determining the effector to memory conversions are largely unknown in parasitic diseases, the early antigen clearance, appropriate naïve B/T cells activation, and functions are some known prerequisites (59–62). Studies on viral and leishmanial antigens suggest that repetitive and persistent antigen exposure inhibits the conversion of effector T cells to their memory phenotypes further supporting the requirements of early antigen clearance after primary exposure for a better memory pool (63, 64). A few studies also suggest that the nature and duration of signals, which T cells receive from the APCs determine the numbers of central memory cells during an early immune response (65, 66). However, once central memory is established its behavior and effector properties remain grossly similar to precursor effector cells. Though the functional properties of CD4^+^ T cells memory phenotypes, expressing high levels of both CCR7 and CD62L receptors on their surface, remain the same as of effector T cells at the time of primary antigen exposure with *Leishmania* antigens (67), the poor conversion of effector T cells into their memory phenotypes remains a major obstacle in achieving a durable immunity (68). We observed increased numbers of CCR7^+^ and CD62L^+^ CD4+ T cells in anti-CD300a antibodies treated mice, which was significant at the later stages of infection (D14 and D21). It provides strong indicative evidence for the crucial role of CD300a in the development of immunologic memory against *Leishmania* antigens.

**Figure 7:**
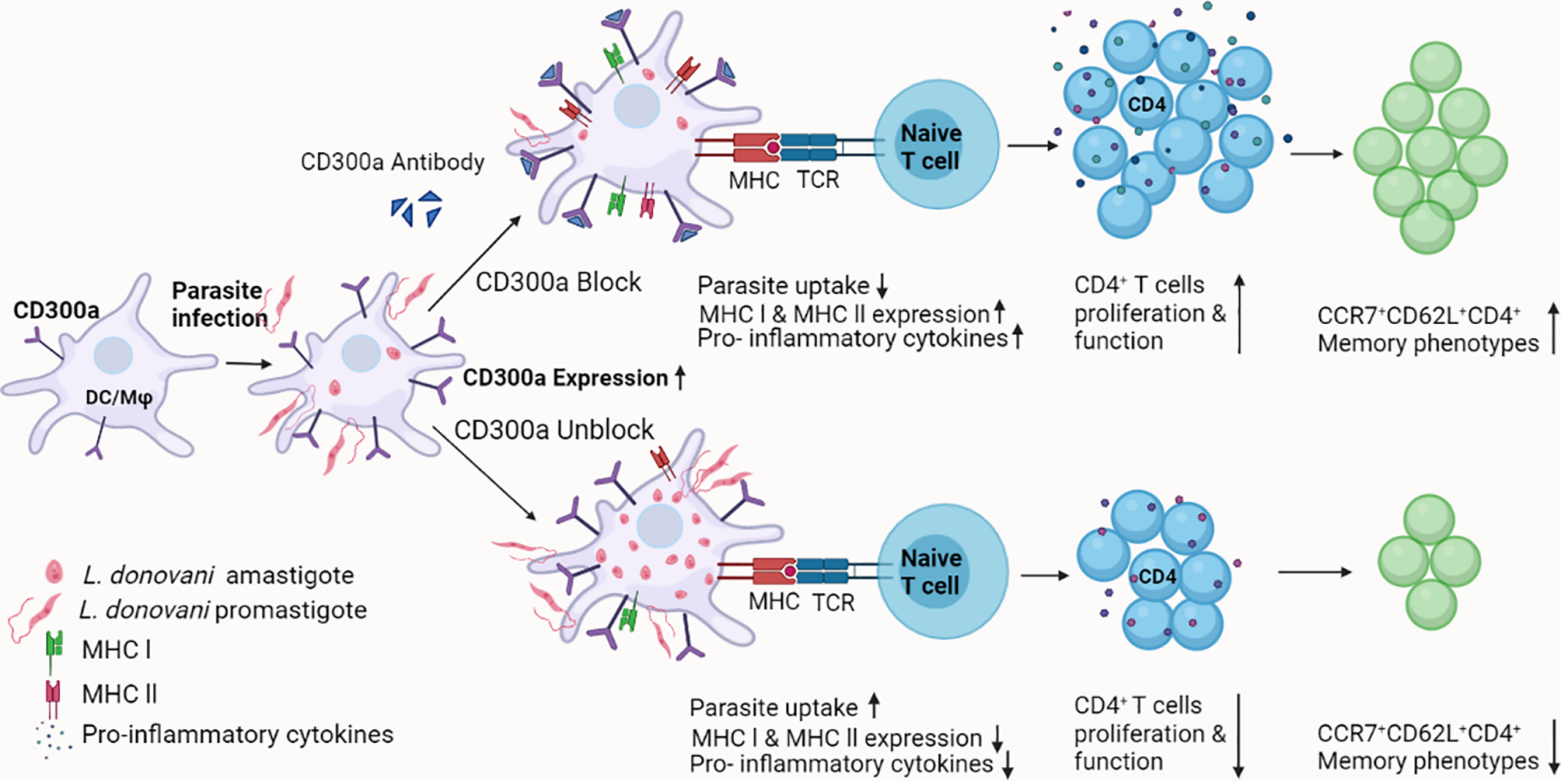
A schematic model for the role of CD300a receptor in parasitic infectivity, APCs and T cells effector functions, and memory generation during *Leishmania* infection. Based on observations made in the current study, it may be surmised that the parasite induces CD300a receptors on the host’s phagocytic and antigen presenting cells to counter the defense machinery, which eventually facilitates the survival of parasites. The blocking of CD300a receptors increases APCs function and promotes antigen specific T cells activation and differentiation required to establish early protective immunity. Thus, strategies involving CD300a receptor manipulation may prove advantageous to curb the survival of *Leishmania* in their mammalian host and also can boost antigen-specific immune responses.

Taken together, our results suggest that CD300a signaling is detrimental to the quality and magnitude of phagocytic, APCs, and T cells effector functions in experimental visceral leishmaniasis caused by *L. donovani*. Further, these findings provided a novel way to increase the effector functions of CD4^+^ T cells that may result in increased conversion of antigen experienced T cells into their memory phenotypes during early immune responses against parasitic antigens (**Figure 7**). More in-depth studies are required to elucidate the role of CD300a signaling abrogation by anti-CD300a antibodies/agents in the establishment of vaccine induced cell-mediated protective immunity against intracellular pathogens, in general, and against *Leishmania*, in particular. It could pave the way to develop better vaccines against intracellular pathogens.

## Data Availability Statement

The original contributions presented in the study are included in the article/**Supplementary Material**. Further inquiries can be directed to the corresponding author.

## Ethics Statement

The animal study was reviewed and approved by Institutional Animal Ethics Committee (IAEC), Institute of Science, BHU, Varanasi.

## Author Contributions

RS, AA, AR, and BM: designed and performed the major experiments like flow cytometry, IHC, immunoblotting, qPCR etc. ShS and NKS: performed mice and parasites/cells culture-related works. NS, VK, DK, and PD: assisted in conceptualization and design of the study. SaS and AM: assisted in statistical analysis and MS writing. RKS: conceived, designed, directed, wrote and supervised the complete study. All authors contributed to the article and approved the submitted version.

## Funding

This work is an output of a project funded to RKS by the Department of Biotechnology; New Delhi (No. BT/PR24210/MED/15/172/2017).

## Conflict of Interest

The authors declare that the research was conducted in the absence of any commercial or financial relationships that could be construed as a potential conflict of interest.

## Publisher’s Note

All claims expressed in this article are solely those of the authors and do not necessarily represent those of their affiliated organizations, or those of the publisher, the editors and the reviewers. Any product that may be evaluated in this article, or claim that may be made by its manufacturer, is not guaranteed or endorsed by the publisher.

## Supplementary Material

Supplementary Figure 1 The numbers of amastigotes per 100 macrophages **(A)** and BMDCs **(B).** The infected cells were incubated up to 48h in a CO_2_ incubator and intracellular amastigotes were counted at 4h, 12h, 24h, and 48h post infection. **(C)** The proliferation rates of anti-CD300a antibodies treated parasites were found comparable to untreated parasites. **(D)** Duramycin, a PE blocker, reduced parasite uptake by the macrophages (p=0.018) and dendritic cells (p=0.023) and it did not affect parasite growth (inset of the panel **D**).

Supplementary Figure 2 The nitric oxide (NO), measured in terms of nitrite produced by the cells and mRNAs expression levels of pro and anti-inflammatory cytokines in infected macrophages and BMDCs after CD300a blocking The CD300a receptor blocking significantly enhanced the production levels of NO (and proinflammatory cytokines and reduced the levels of anti-inflammatory cytokines in macrophages and BMDCs **(A–C)**.

Supplementary Figure 3 The proliferation of antigen experienced CD4^+^ and CD8^+^ T cells co-cultured with macrophage in the presence and absence of anti-CD300a antibodies. The CD300a receptor blocking significantly enhanced their proliferation.

Supplementary Figure 4 The mRNAs expression levels of pro and anti-inflammatory cytokines, and MHC genes in the spleen of infected mice. The CD300a receptor blocking in *L. donovani* infected mice induced the IFN-γ and IL-12 **(A, B)** while inhibited IL-4 and IL-10 mRNAs expression levels **(C, D)** in their spleen. The CD300a blocking also induced the expression of both MHC I and MHC II genes in the spleen of parasite infected animals **(E, F)**.

Supplementary Figure 5 The liver granuloma formation in anti-CD300a antibodies treated mice. The arrows indicate the macrophage infiltration i.e., the granulomatous portion in the tissue sections. The CD300a blocking significantly reduced the granuloma formation at the later days of infection.
