# Supplementary figures and images for: CD300a Receptor Blocking Enhances Early Clearance of Leishmania donovani From Its Mammalian Host Through Modulation of Effector Functions of Phagocytic and Antigen Experienced T Cells
Source: Front Immunol. 2022 Jan 18;12:793611. doi: 10.3389/fimmu.2021.793611 (PMC8803664; doi:10.3389/fimmu.2021.793611)

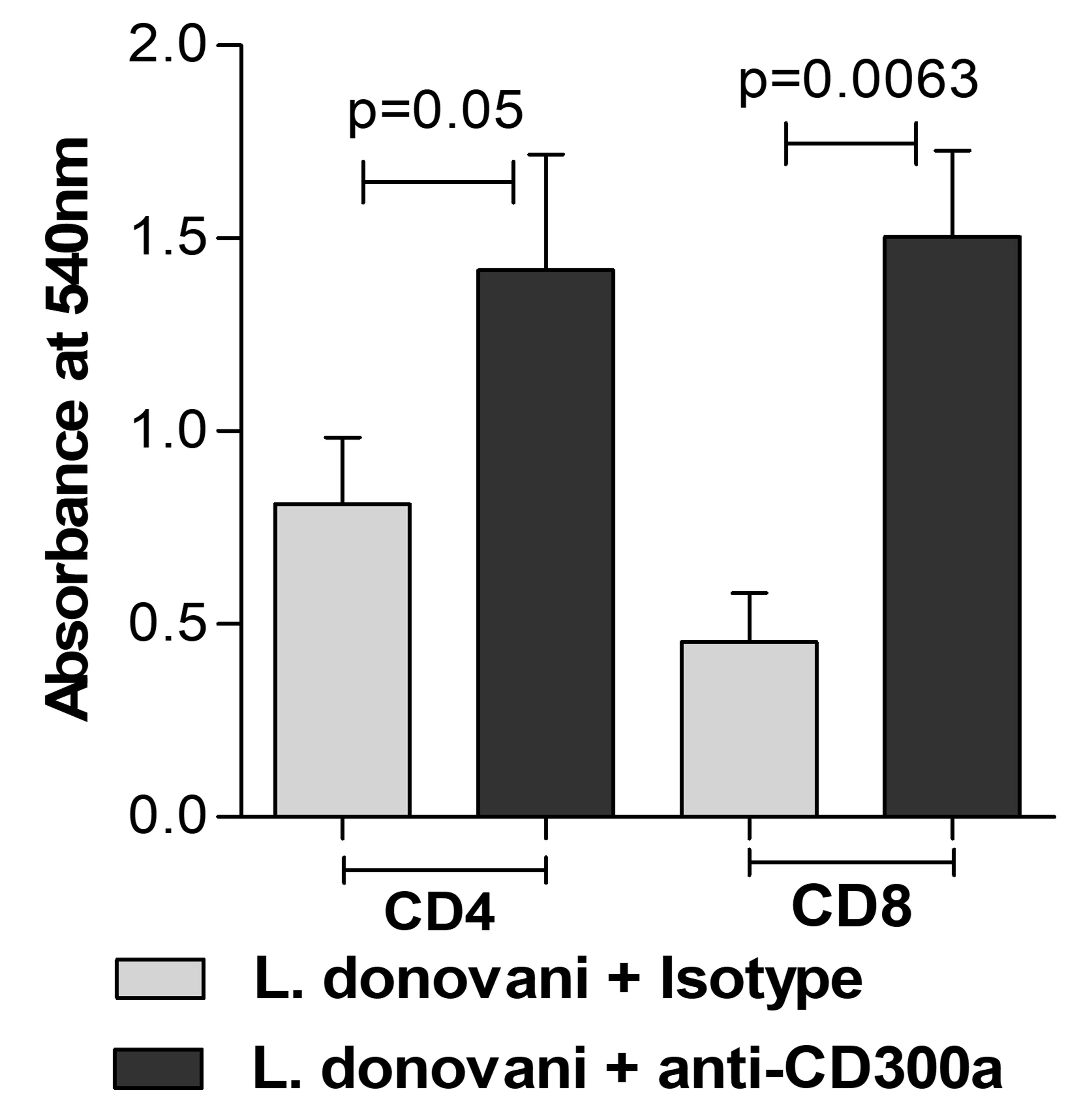

Supplement: Supplementary Figure 3 — The proliferation of antigen experienced CD4+ and CD8+ T cells co-cultured with macrophage in the presence and absence of anti-CD300a antibodies. The CD300a receptor blocking significantly enhanced their proliferation. [file Image_3.tif]

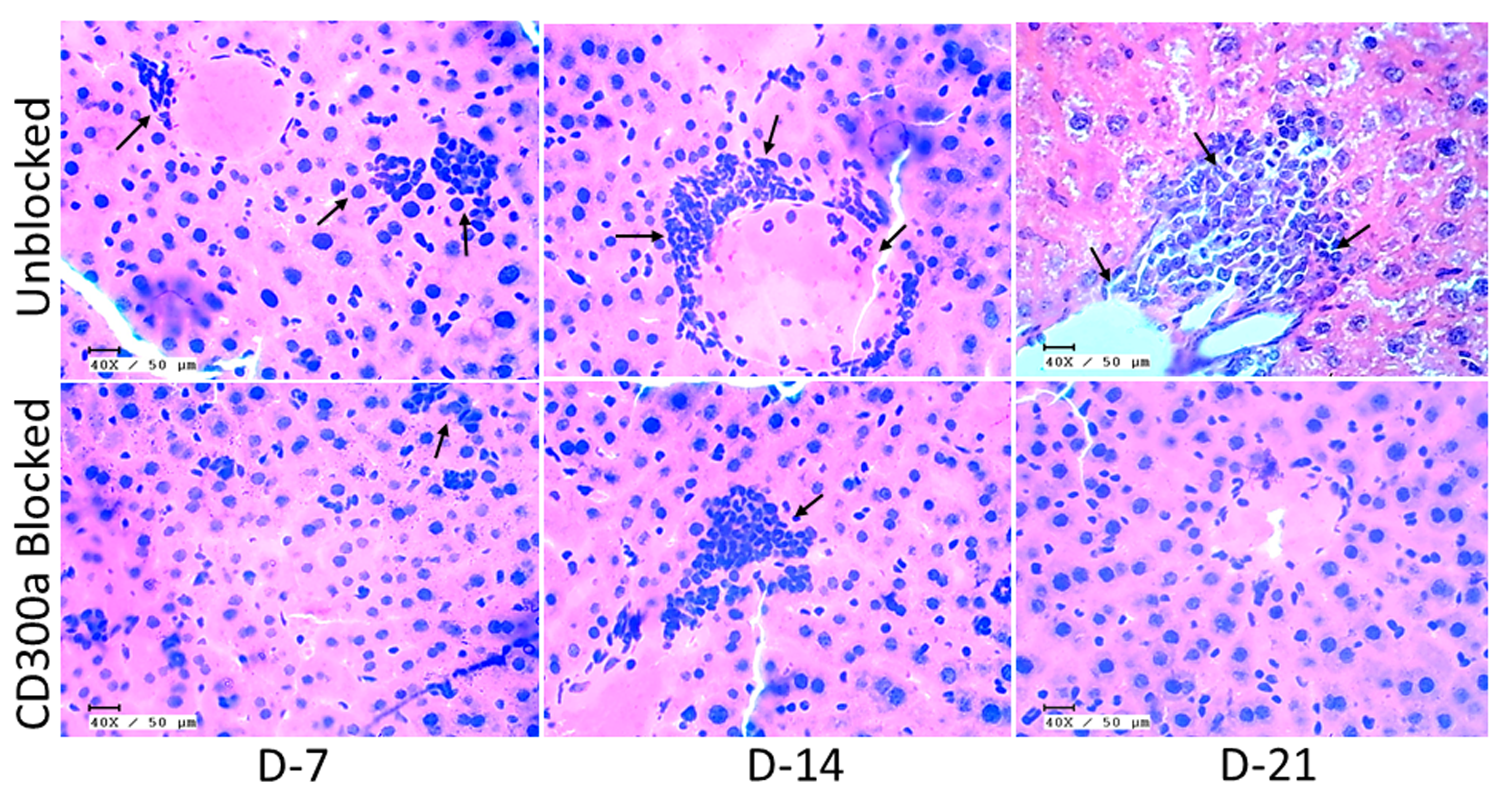

Supplement: Supplementary Figure 5 — The liver granuloma formation in anti-CD300a antibodies treated mice. The arrows indicate the macrophage infiltration i.e., the granulomatous portion in the tissue sections. The CD300a blocking significantly reduced the granuloma formation at the later days of infection. [file Image_5.tif]
