# Supplementary material for: CD300a Receptor Blocking Enhances Early Clearance of Leishmania donovani From Its Mammalian Host Through Modulation of Effector Functions of Phagocytic and Antigen Experienced T Cells
Source: Front Immunol. 2022 Jan 18;12:793611. doi: 10.3389/fimmu.2021.793611 (PMC8803664; doi:10.3389/fimmu.2021.793611)
Supplement: Supplementary file 6 [file Table_1.docx]

**Supplementary Table 1**

| **S.No.** | **Primer Name** | **Primer Sequence** |
| --- | --- | --- |
| 1 | GAPDH | **Forward primer** ACACAGTAGACTCCACG  **Reverse primer** TCCAGTATGACTCCACTCAG |
| 2 | IL-12 | **Forward primer** TTGATGGCCTGGAACTCTGT  **Reverse primer** TTCCACAACAAGAGGGAGCT |
| 3 | IFN-γ | **Forward primer** TGAACGCTACACACTGCATC  **Reverse primer** TCCTTTTGCCAGTTCCTCCA |
| 4 | IL-10 | **Forward primer** ACTCTTCACCTGCTCCACTG  **Reverse primer** CCTGGGTGAGAAGCTGAAGA |
| 5 | IL-4 | **Forward primer** ACCTTGGAAGCCCTACAGAC  **Reverse primer** TCTCGAATGTACCAGGAGCC |
| 7 | CD300a | **Forward primer** TCATCCGTGGCATTCAACTC  **Reverse primer** TCCTTTTCTGGGCTTCTGGA |
| 8 | MHC-I | **Forward primer** GAAGTGGATTACGGAGGGGT  **Reverse primer** TGCTCACTCGAAGGATGTCC |
| 9 | MHC-II | **Forward primer** GAAGTGGATTACGGAGGGGT  **Reverse primer** TGCTCACTCGAAGGATGTCC |
| 10 | TNF- a | **Forward primer** TGTCTTTGAGATCCATGCCG  **Reverse primer** GGCACAGGGTCATCATCAAA |
